# Lactoferrin Ameliorates Ovalbumin-Induced Asthma in Mice through Reducing Dendritic-Cell-Derived Th2 Cell Responses

**DOI:** 10.3390/ijms232214185

**Published:** 2022-11-16

**Authors:** Chi-Chien Lin, Kai-Cheng Chuang, Shih-Wei Chen, Ya-Hsuan Chao, Chih-Ching Yen, Shang-Hsun Yang, Wei Chen, Kuang-Hsi Chang, Yu-Kang Chang, Chuan-Mu Chen

**Affiliations:** 1 Department of Life Sciences, Ph.D. Program in Translational Medicine, National Chung Hsing University, Taichung 402, Taiwan; 2 Institute of Biomedical Sciences, National Chung Hsing University, Taichung 402, Taiwan; 3 Department of Medical Research, China Medical University Hospital, Taichung 40447, Taiwan; 4 Department of Pharmacology, College of Medicine, Kaohsiung Medical University, Kaohsiung 80708, Taiwan; 5 Department of Otolaryngology, Tungs’ Taichung MetroHarbor Hospital, Taichung 435, Taiwan; 6 Department of Internal Medicine, China Medical University Hospital, College of Health Care, China Medical University, Taichung 404, Taiwan; 7 Department of Physiology, Institute of Basic Medical Sciences, College of Medicine, National Cheng Kung University, Tainan 701, Taiwan; 8 Division of Pulmonary and Critical Care Medicine, Chia-Yi Christian Hospital, Chiayi 600, Taiwan; 9 Department of Medical Research, Tungs’ Taichung MetroHarbor Hospital, Taichung 435, Taiwan; 10 The iEGG and Animal Biotechnology Center, The Rong Hsing Research Center for Translational Medicine, National Chung Hsing University, Taichung 402, Taiwan

**Keywords:** lactoferrin, airway hyperresponsiveness, pulmonary inflammation, allergy, asthma

## Abstract

Asthma is a chronic respiratory disease with symptoms such as expiratory airflow narrowing and airway hyperresponsiveness (AHR). Millions of people suffer from asthma and are at risk of life-threatening conditions. Lactoferrin (LF) is a glycoprotein with multiple physiological functions, including antioxidant, anti-inflammatory, antimicrobial, and antitumoral activities. LF has been shown to function in immunoregulatory activities in ovalbumin (OVA)-induced delayed type hypersensitivity (DTH) in mice. Hence, the purpose of this study was to investigate the roles of LF in AHR and the functions of dendritic cells (DCs) and Th2-related responses in asthma. Twenty 8-week-old male BALB/c mice were divided into normal control (NC), ovalbumin (OVA)-sensitized, and OVA-sensitized with low dose of LF (100 mg/kg) or high dose of LF (300 mg/kg) treatment groups. The mice were challenged by intranasal instillation with 5% OVA on the 21st to 27th day after the start of the sensitization period. The AHR, cytokines in bronchoalveolar lavage fluid, and pulmonary histology of each mouse were measured. Serum OVA-specific IgE and IgG1 and OVA-specific splenocyte responses were further detected. The results showed that LF exhibited protective effects in ameliorating AHR, as well as lung inflammation and damage, in reducing the expression of Th2 cytokines and the secretion of allergen-specific antibodies, in influencing the functions of DCs, and in decreasing the level of Th2 immune responses in a BALB/c mouse model of OVA-induced allergic asthma. Importantly, we demonstrated that LF has practical application in reducing DC-induced Th2 cell responses in asthma. In conclusion, LF exhibits anti-inflammation and immunoregulation activities in OVA-induced allergic asthma. These results suggest that LF may act as a supplement to prevent asthma-induced lung injury and provide an additional agent for reducing asthma severity.

## 1. Introduction

Asthma is a serious respiratory disease affecting more than 300 million people worldwide [1]. Asthma involves chronic airway inflammation, expiratory airflow narrowing, and airway hyperresponsiveness (AHR), causing respiratory symptoms characterized by tachypnea, cough, and chest pain [2]. The contributing factors of asthma are complex and associated with interactions between genes and environments [3]. The immune responses of asthma are allergic sensitization, dendritic cell (DC) activation, and cellular immunity, which are involved in mediating inflammatory cells and mediators in respiratory tracts [4]. Allergens induce IgE secretion and trigger eosinophils, basophils, and mast cells to secrete mediators of allergenic inflammation [5]. Asthma is a highly heterogeneous disease that makes clinical diagnosis difficult. Although many drugs and nonpharmacological treatment strategies have been developed, there are still many asthma patients whose symptoms are poorly controlled or continue to worsen. Therefore, the development of new treatment methods is still urgently needed.

Endotypes of asthma are defined as type-2 high (T2), type-2 low (non-T2), or a mixture of T2 and non-T2 on the basis of immune-inflammatory pathways [6]. Allergic asthma is typically classified as a T2 immune-mediated condition and is accompanied by Th2-related cytokine production, eosinophilic airway inflammation, and circulating IgE secretion [7]. After allergen sensitization, DCs engulf allergens and present small peptides to naïve T cells. The stimulated naïve T cells differentiate into Th1, Th2, Th9, Th17, and regulatory T (T_reg_) cells and produce Th1-related cytokines (IFN-γ and IL-12), Th2-related cytokines (IL-4, IL-5, and IL-13), Th9-related cytokines (IL-9), Th17-related cytokines (IL-17A and F), and T_reg_-related cytokines (TGF-β and IL-10), and then induce asthmatic inflammation in the airway [4,8,9,10]. These cytokines then enhance IgE production, eosinophil accumulation, mast cell proliferation, mucus hypersecretion, and AHR [4,11].

Lactoferrin (LF) is a well conserved, monomeric signal polypeptide chain glycoprotein with an 80 kDa molecular weight and 690 amino acid residues. LF has the ability to conjugate and transfer Fe^3+^ ions and is referred to as a part of the transferrin family, known as lactotransferrin [12,13,14]. LF has multiple physiological functions, including antioxidant, anti-inflammatory, antibacterial, antifungal, antiviral, antiparasitic, and antitumoral activities [15]. LF is a modulator secreted by cells and has functions in innate and adaptive immunity in mammals [16]. LF has profound abilities in adaptive immune responses, such as promoting T-cell differentiation and B-cell maturation [17,18]. Additionally, LF is a postulated ligand for the mannose receptor and has immunoregulatory activities in ovalbumin (OVA)-induced delayed type hypersensitivity (DTH) in mice [19]. Although many physiological functions of LF have been reported, the receptors and immune regulatory mechanisms of LF are still unknown [16].

Asthma is a classical Th2 disease because Th2-related cytokines play predominant roles in AHR [11]. The level of production of IgE, IL-4, IL-5, IL-13, and IFN-γ is related to the severity of asthmatic symptoms in T2 asthma [20]. In this study, we first aimed to investigate the roles of LF in AHR and Th2-related responses after OVA-induced asthma in mice. We further investigated the effects of LF on DC functions after OVA-sensitization in mice. The intention of this study was to explore the therapeutic effects of LF in asthma by evaluating the level of OVA-induced Th2 immunity in a BALB/c mouse model.

## 2. Results

### 2.1. LF Improved OVA-Induced AHR and Pulmonary Inflammation in BALB/c Mice

**Figure 1:**
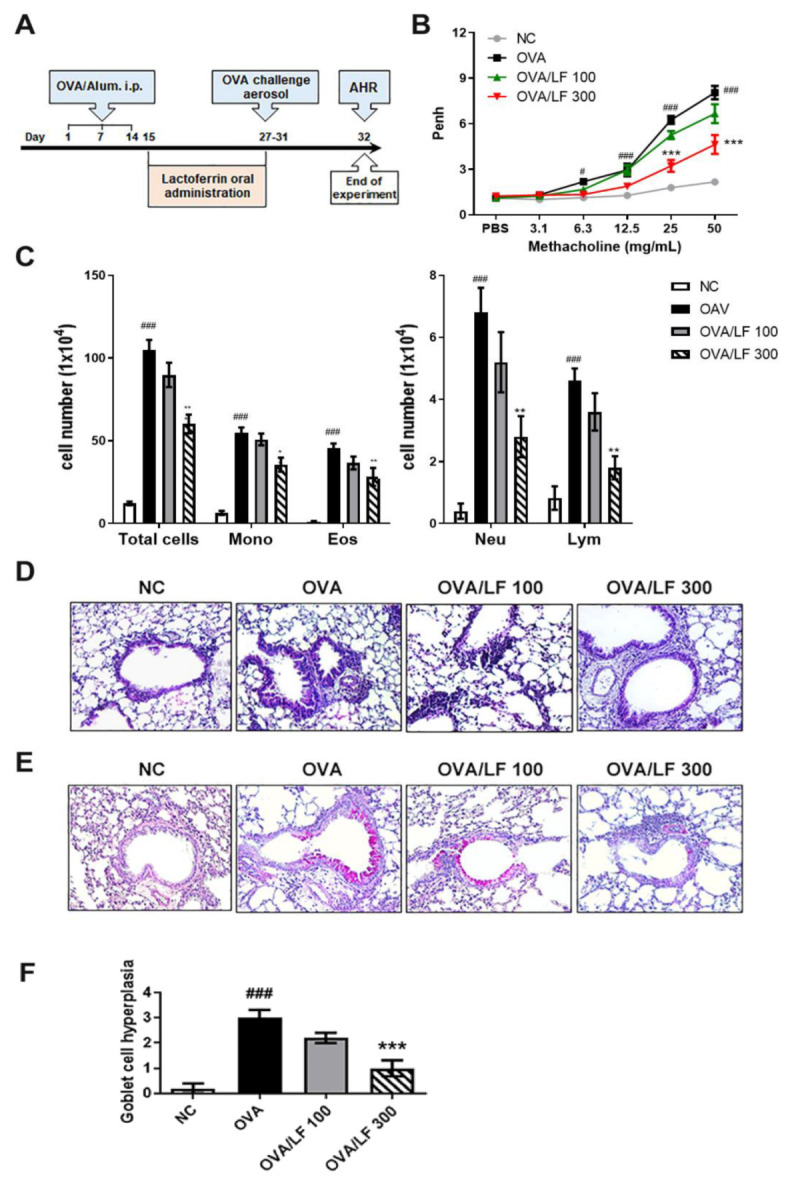
Effect of lactoferrin on AHR and airway inflammation. (**A**) Experimental procedure of the OVA-induced AHR animal experiment. Mice were randomly divided into 4 groups (5 mice/group): normal control (NC), ovalbumin (OVA)-sensitized, OVA-sensitized and low-dose lactoferrin (OVA + LF 100 mg/kg), and OVA-sensitized and high-dose lactoferrin (OVA + LF 300 mg/kg). The NC group was not sensitized with OVA and treated with lactoferrin. Mice in the other groups were i.p. injected with 20 μg OVA containing 2% alum on the 1st, 7th, and 14th days. On the 15th to 27th days, mice in the OVA/LF 100 and OVA/LF 300 groups were administered 100 or 300 mg/kg lactoferrin orally every day. On the 27th to 31st day, mice in the OVA, OVA/LF 100, and OVA/LF 300 groups were intranasally challenged with 5% OVA. Mice were sacrificed on the 32nd day for experiments. (**B**) The effect of lactoferrin on the degree of bronchoconstriction and enhanced pause (Penh) values were determined. On the 32nd day, mice inhaled increasing doses of methacholine (Mch; 3.125–50 mg/mL). Penh levels represent the degree of AHR. (**C**) Inflammatory cell counts in the BALF. The numbers of monocytes, eosinophils, neutrophils, and lymphocytes in the BALF were determined by Diff-Quik reagent staining. Histopathological examination of pulmonary tissues was performed by (**D**) H&E staining and (**E**) periodic acid-Schiff staining. (**F**) Quantification of goblet cell number in the slide using a semiquantitative scoring system. The pathogenic scores of goblet cell hyperplasia were determined according to the modified 5-point scoring system (grade 0–4) and were expressed by score according to the percentage of the goblet cells in the epithelium: 0 (no goblet cells), 1 (<25%), 2 (25–50%), 3 (51–75%), and 4 (>75%). The data are expressed as the mean ± S.E.M. values (*n* = 5). ^#^ *p* < 0.05, ^###^ *p* < 0.001 vs. NC group; * *p* < 0.05, ** *p* < 0.01, *** *p* < 0.001 vs. OVA group. Mono: monocytes; Eos: eosinophils; Neu: neutrophils; Lym: lymphocytes. Images were captured with bright-field microscopy (magnification 400×).

To investigate the effects of LF on OVA-induced AHR, we established an OVA-induced asthmatic model in mice. The experimental procedure of OVA sensitization and challenge and LF treatment is shown in Figure 1A. The change in breathing patterns in the plethysmograph box pressure was monitored, and the impulse and maximum pressure changes of inspiration and expiration were calculated as value-enhanced pause (Penh) [21,22]. Penh was used to estimate changes in pulmonary function after methacholine (Mch) exposure, as shown in Figure 1B. Compared with that of the normal control (NC) group, Penh was increased in the OVA group following inhalation of 6.3 mg/mL to 50 mg/mL Mch, whereas Penh was significantly decreased in the mice administered 300 mg/kg LF after inhalation of 25 and 50 mg/mL Mch. OVA inhalation significantly increased the numbers of inflammatory cells, including total cells, monocytes, eosinophils, neutrophils, and lymphocytes, in the bronchoalveolar lavage fluid (BALF) in the OVA group compared with the NC group, but oral administration of 300 mg/kg LF attenuated the increasing numbers of inflammatory cells in the BALF (Figure 1C). The effects of LF on the histopathological changes were determined with H&E staining in the pulmonary tissues (Figure 1D). The degree of inflammatory cell infiltration in the peribranchial and perivascular areas was notably observed in the OVA alone group compared with the NC and OVA/LF groups (Figure 1D). The level of goblet cell hyperplasia was examined with periodic acid-Schiff staining in pulmonary tissues. Compared with those of the OVA group, the numbers of goblet cells determined in violet-colored regions in the bronchial airway tissues were reduced in the mice treated with 300 mg/kg LF (*p* < 0.001; Figure 1E,F). Collectively, these results showed that LF may decrease OVA-induced allergic airway inflammation in mice with OVA-induced asthma.

### 2.2. LF Suppressed the Production of OVA-Induced Typical Th2 Cytokines and Increased Anti-Inflammatory Cytokines in the BALF

**Figure 2:**
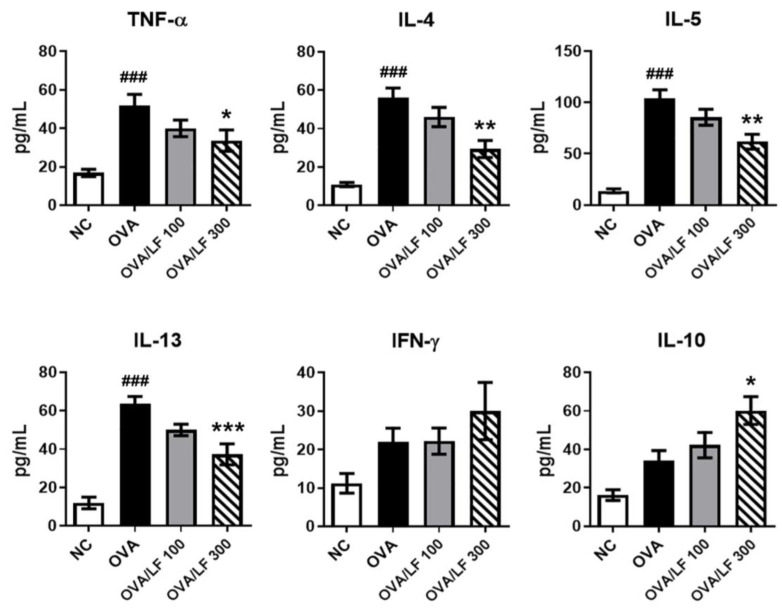
Effects of lactoferrin on Th2 cytokine production in BALF. The levels of TNF-α, IL-4, IL-5, IL-13, IFN-γ, and IL-10 in the BALF were detected by ELISAs. The data are expressed as the mean ± S.E.M. values (*n* = 5). ^###^ *p* < 0.001 vs. the NC group; * *p* < 0.05, ** *p* < 0.01, *** *p* < 0.001 vs. the OVA group. NC: Normal control; OVA: ovalbumin; OVA/LF 100: OVA + lactoferrin 100 mg/kg; OVA/LF 300: OVA + lactoferrin 300 mg/kg.

Asthmatic inflammation-related cytokines include TNF-α, Th2 cytokines (L-4, IL-5 and IL-13), Th1 cytokines (IFN-γ and IL-12), and anti-inflammatory cytokines (IL-10) [20]. We further examined the expression of asthmatic inflammation-related cytokines in the BALF. Compared with that in the NC group, the secretion of TNF-α, IL-4, IL-5, and IL-13 was increased in the OVA group (Figure 2). Importantly, in the 300 mg/kg high-dose LF-treated group, the secretion of TNF-α, IL-4, IL-5, and IL-13 was significantly reduced compared with that in the OVA group. In addition, we observed that 300 mg/kg LF treatment significantly increased the secretion of IL-10 compared with that in the OVA group (*p* < 0.05). However, there was no significant difference in IFN-γ among all groups. These results suggested that 300 mg/kg LF treatment may have the potential to modulate Th2-mediated cytokine and anti-inflammatory cytokine expression in the lungs of OVA-induced asthmatic mice.

### 2.3. LF Regulated OVA-Specific IgG1 and IgE Secretion in the Serum

**Figure 3:**
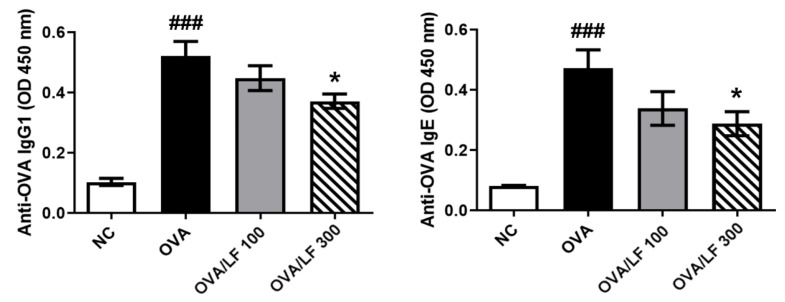
Effects of lactoferrin on OVA-specific IgG1 and IgE production in serum. The levels of OVA-specific IgG1 and IgE were detected by ELISAs. The data are expressed as the mean ± S.E.M. values (*n* = 5). ^###^ *p* < 0.001 vs. the NC group; * *p* < 0.05 vs. the OVA group. NC: Normal control; OVA: ovalbumin; OVA/LF 100: OVA + lactoferrin 100 mg/kg; OVA/LF 300: OVA + lactoferrin 300 mg/kg.

The levels of OVA-specific IgG and IgE are important parameters in OVA-induced AHR [23]. We next analyzed the expression of OVA-related IgG1 and lgE in different groups of mice. The levels of OVA-specific IgE and IgG1 in the OVA group were significantly increased compared with those in the NC group (*p* < 0.001; Figure 3). Compared with the OVA treatment, treatment with 300 mg/kg LF significantly decreased the levels of total OVA-specific IgE and IgG1 (*p* < 0.05; Figure 3). These results demonstrated that LF treatment regulated the secretion of OVA-specific IgG1 and IgE in mice with OVA-induced asthma.

### 2.4. LF Decreased OVA-Specific Th2 Responses in the Spleen

**Figure 4:**
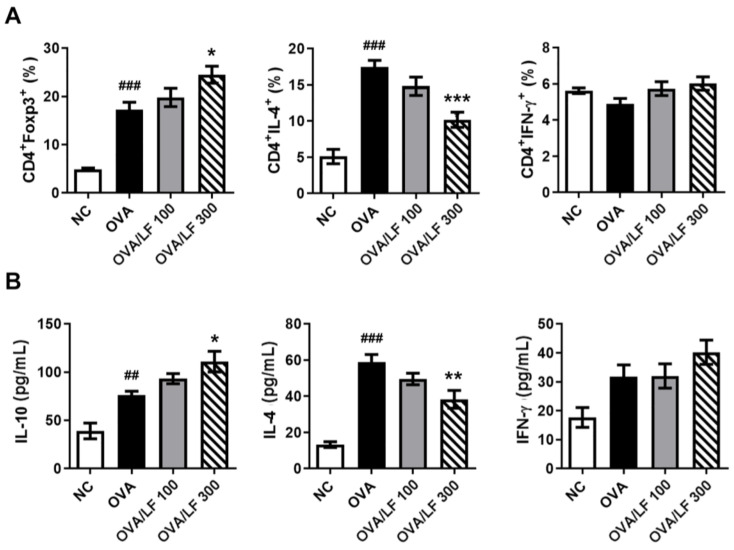
Lactoferrin increased the proportion of CD4^+^Foxp3^+^ Tregs and decreased IL-4-producing T cells in the splenocyte population. (**A**) The levels of OVA-specific Foxp3, IL-4, and IFN-γ in splenocytes were determined by flow cytometry with specific antibodies. (**B**) The levels of IL-10, IL-4, and IFN-γ secreted by splenocytes were detected by ELISAs. The data are expressed as the mean ± S.E.M. values (*n* = 5). ^##^ *p* < 0.01, ^###^ *p* < 0.001 vs. the NC group; * *p* < 0.05, ** *p* < 0.01, *** *p* < 0.001 vs. the OVA group. NC: Normal control; OVA: ovalbumin; OVA/LF 100: OVA + lactoferrin 100 mg/kg; OVA/LF 300: OVA + lactoferrin 300 mg/kg.

DCs play crucial roles in polarizing CD4^+^ T cells to Th1, Th2, and T_reg_ cells. Allergic asthma is predominant in Th2 responses [24]. We further investigated the proliferation of spleen cells under in vitro stimulation with OVA antigen. To distinguish the effects of LF in the population of T cells in OVA-induced asthma, we used Foxp3, IL-4, INF-γ, and CD4 antibodies to evaluate the differentiation of T cells. Foxp3^+^ T_reg_ cells regulate Th2 responses and inhibit allergy and asthma. IL-4^+^ and INF-γ^+^ T_reg_ cells are responsible for Th2 and Th1 responses, respectively. In Figure 4A and Figure S1, we observed that the number of CD4^+^/Foxp3^+^ Treg cells in the splenocyte population increased in the OVA-treated group compared with the NC group (*p* < 0.05). Furthermore, we observed that the number of CD4^+^/Foxp3^+^ T_reg_ cells in the splenocyte population increased in the 300 mg/kg LF-treated group compared with the OVA group (*p* < 0.05), but the number of CD4^+^/IL4^+^ T cells decreased (*p* < 0.001). IL-4, IFN-γ, and IL-10 are attributed to Th2, Th1, and anti-inflammatory cytokines. The IL-4 level was significantly decreased in the supernatants of splenocytes in the high-dose LF-treated group (*p* < 0.01), whereas the IL-10 level was significantly increased in the 300 mg/kg LF-treated group (*p* < 0.05) compared with the OVA group (Figure 4B). However, there were no significant differences in the number of CD4^+^/IFN-γ^+^ T cells or the expression of IFN-γ among all groups. These results showed that LF could inhibit Th2 responses in OVA-induced asthma in mice.

### 2.5. LF Downregulated the Surface Molecules CD80 and CD86 in DCs in the Spleen of OVA-Treated Mice

**Figure 5:**
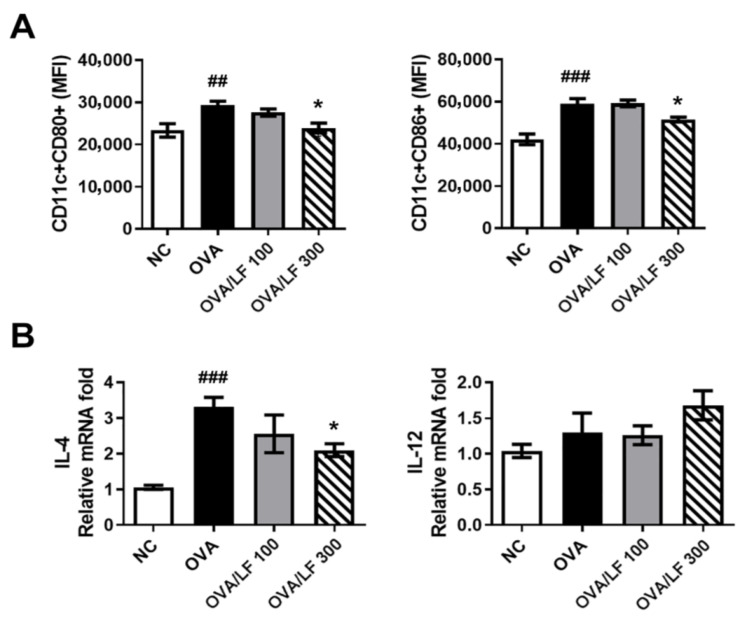
Lactoferrin downregulated the expression of CD80, CD86, IL-4, and IL-12 cytokines in DCs. (**A**) The levels of CD80 and CD86 expression in CD11c+ DC cells were determined by flow cytometry with specific antibodies. (**B**) The levels of IL-4 and IL-12 production in CD11c+ DC cells were detected by qRT-PCR. The data are expressed as the mean ± S.E.M. values (*n* = 5). ^##^ *p* < 0.01, ^###^ *p* < 0.001 vs. the NC group; * *p* < 0.05 vs. the OVA group. NC: Normal control; OVA: ovalbumin; OVA/LF 100: OVA + lactoferrin 100 mg/kg; OVA/LF 300: OVA + lactoferrin 300 mg/kg.

DCs are principal antigen-presenting cells that play an important role in the regulation of T-cell mediated immune responses in an OVA-induced asthma model. Thus, we further explored the activation of DCs obtained from the splenocytes in different groups. On the 33rd day after the first immunization, mice were sacrificed and splenocyte suspensions were prepared. The expression of the costimulatory molecules CD80 and CD86 on CD11c^+^ DCs was examined by flow cytometry. In Figure 5A and Figure S2, the expression of CD80 and CD86 in DCs was significantly increased in the OVA group compared with the NC group. In contrast, treatment with 300 mg/kg LF significantly downregulated the expression of CD80 and CD86 in DCs compared with that in the OVA group (*p* < 0.05). Consistently, the level of IL-4, which plays an essential role in the initiation of Th2 responses in DCs, in the OVA group was significantly increased (Figure 5B). IL-4 expression in the 300 mg/kg LF-treated group was significantly downregulated (*p* < 0.05; Figure 5B). However, the level of the Th1 cytokine IL-12 was not different among all groups. These results demonstrated that LF decreased DC maturation and IL-4 production in DCs from mice with OVA-induced asthma.

### 2.6. LF Decreased the Capacity of DCs to Stimulate OVA-Specific Th2-Cell Responses In Vitro

**Figure 6:**
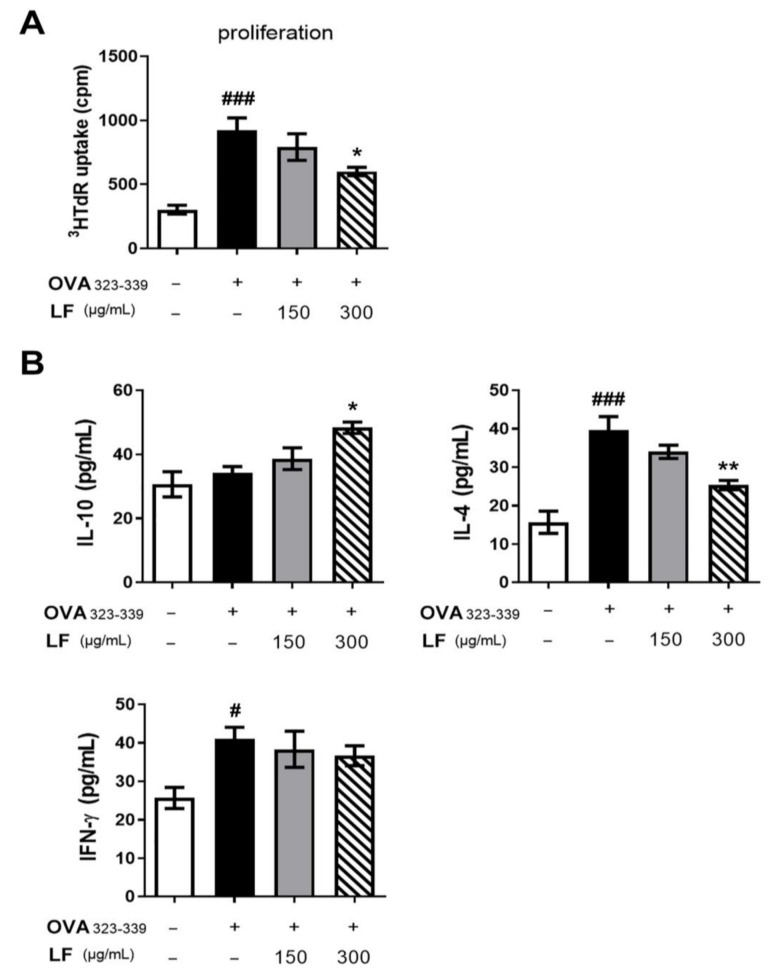
Lactoferrin inhibits OVA-specific T-cell activation induced by OVA_323–339_ (OVAP2) peptide-loaded DCs in vitro. BMDCs from C57BL/6 mice were incubated with or without lactoferrin (150 or 300 μg/mL) and then pulsed with OVA323–339 (OVAP2) peptide for 24 h. After incubation, the isolated DCs and OT-II CD4^+^ T cells were cocultured with DCs:T at a ratio of 1:10 for 72 h. (**A**) The proliferation rate of cocultured cells was evaluated by [^3^H] thymidine uptake. (**B**) The levels of IL-10, IL-4, and IFN-γ production in cocultured cells were detected by ELISAs. The data are expressed as the mean ± S.E.M. values (*n* = 5). ^#^ *p* < 0.05, ^###^ *p* < 0.001 vs. the NC group; * *p* < 0.05, ** *p* < 0.01 vs. the OVAP2 group. OVA_323–329_: OVAP2; LF: lactoferrin.

To further understand the effects of LF on DC functions in OVA-induced asthma, we investigated the effects of LF on the ability of DCs to induce OVA-specific T-cell responses. We established an in vitro system by treating BMDCs from C57BL/6 mice with OVA antigen. OVA_323–339_ (OVAP2) peptide-loaded immature mouse DCs were pretreated in the presence or absence of 150 or 300 μg/mL LF, and the capacity of DCs to stimulate allogeneic OVA-specific OT-II T cells was surveyed. As shown in Figure 6A, the OVA_323–339_ (OVAP2) peptide-treated BMDCs exhibited increased proliferation in T cells (*p* < 0.001). However, proliferation was significantly reduced after 300 μg/mL LF treatment (*p* < 0.05). In addition, the level of IL-10 was increased (*p* < 0.05), but the level of IL-4 was decreased (*p* < 0.01) in the 300 μg/mL LF-treated group (Figure 6B). In contrast to the results for IL-4, the level of IFN-γ was not significantly different between the OVAP2 and LF groups (Figure 6B). Overall, the results indicated that LF impeded the ability of DCs to prime Th2-cell responses in OVA-induced asthma in mice.

## 3. Discussion

In this study, we demonstrated that LF had protective effects in asthma. We also demonstrated that LF influenced the maturation of DCs and decreased the level of Th2 immune responses in allergic asthma. LF may have an effective function in ameliorating AHR, as well as lung inflammation and damage, in asthma. These results indicated that LF could be a supplement for protecting against asthmatic pulmonary damage and could provide an additional reagent for reducing the severity of asthma.

Asthma is a chronic respiratory disease that affects millions of people worldwide [3]. Asthma is still difficult to treat and control in clinical practice. Over 10% and 2.5% of adults and children, respectively, suffer severe asthma with risks of narrowed airflow limitation, exacerbations, hospitalization, and death [2]. Here, we showed the protective effects of LF in asthma. Orally administered LF ameliorated OVA-induced AHR, pulmonary inflammation, Th2-related cytokines, OVA-specific lgG1, and lgE secretion in mice. Furthermore, we found that LF in OVA-induced asthma could suppress DC maturation and OVA-specific Th2 cell responses.

LF is a natural protein in exocrine secretion fluids, such as colostrum, milk, tears, saliva, serum, cerebrospinal, joint, seminal, and vaginal fluids, and has host-defensive and immunoregulatory functions [25,26,27]. LF plays roles in immune responses. For example, LF promotes DC maturation and enhances DC functions to facilitate antigen-specific T-cell differentiation in DTH responses [25,28]. Evidence has shown that LF has functions in DTH, allergies, and asthma [16,25,26,28,29,30,31]. These studies indicated that LF has potential in asthma treatment.

Pulmonary symptoms in asthma are bronchoconstriction, respiratory inflammation, airway remodeling, and AHR [24]. AHR is the most important symptom in asthma and causes airway smooth muscle growth and constriction, respiratory mucus secretion, and inflammation in pulmonary tissue [4,32,33]. Penh has been used to estimate changes in pulmonary function. In Figure 1A,B, we showed that LF administered orally could significantly reduce methacholine-induced AHR after OVA sensitization in mice. Allergen-induced asthma is a complex airway disease that includes innate and adaptive immunity and involves mast cells, type-2 innate lymphoid cells (ILC2s), basophils, eosinophils, alternative activated macrophages (AAMs), DCs, and Th2 cells [20]. Here, we showed that LF treatment could reduce the numbers of asthma-related cells in the airway (Figure 1C). We also showed that lung tissue damage and proinflammatory cell infiltration were ameliorated in the LF-treated groups (Figure 1D,E). Airway goblet cell hyperplasia is associated with asthma [34] and is considered a pathologic feature of mild, moderate, and severe asthma [35]. We found that OVA-induced goblet cell hyperplasia was decreased in the LF-treated groups (Figure 1F). In T2 asthma, the Th2 cytokines IL-4, IL-5, IL-9, IL-13, and IL-25, and the acute proinflammatory cytokines TNF-α, IL-1β, IL-6, and IL-8, subsequently lead to bronchial hyperresponsiveness (BHR), and plasma cells and antibody-producing cells secrete antibodies [20,23]. We demonstrated that LF treatment reduced the secretion of these asthma-related cytokines and antibodies (Figure 2 and Figure 3).

**Figure 7:**
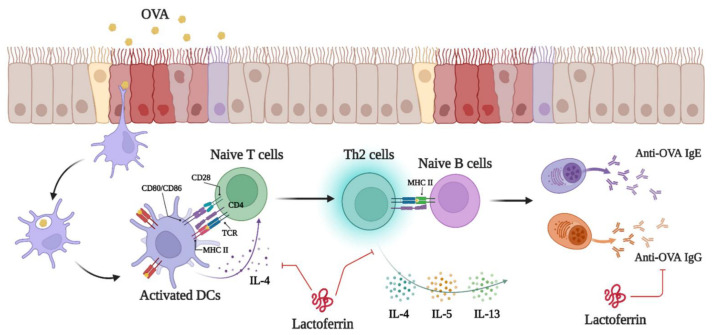
The protective mechanisms of lactoferrin in OVA-induced asthma. Three intraperitoneal injections of OVA and aerosol challenge in mice activated DCs and increased the level of Th2 immune responses to induce allergic asthma and AHR. The protective mechanism of LF in OVA-induced asthma may occur through the inhibition of DC activation and the Th2 cell immune response. This photo was created with BioRender.com software (accessed on 16 August 2022).

There are three types of immunopathology in asthma: (1) eosinophilic asthma (allergic eosinophilic inflammation and nonallergic eosinophilic inflammation), (2) noneosinophilic asthma (paucigranulocytic and type 1 and type 17 neutrophilic inflammation), and (3) mixed granulocytic asthma [3]. Allergic eosinophilic inflammation is DC-Th2-cell-induced respiratory inflammation induced by allergens. Nonallergic eosinophilic inflammation is mast cell- and ILC2-cell-induced respiratory inflammation by pollutants or microbes. Paucigranulocytic is mast-cell-induced BHR caused by pollutants or oxidative stresses. Type 1 and type 17 neutrophilic inflammation are DC-Th1- and Th17-cell-induced respiratory inflammation induced by pollutants, microbes, or oxidative stresses [3]. In our asthmatic animal model, we demonstrated that LF treatment moderated OVA-induced AHR by inhibiting Th2-type immune responses (Figure 4 and Figure 5). In T2 asthma, myeloid dendritic cells (mDCs) present antigens to Th0 cells. Antigen-presented Th0 cells then differentiate into Th2 cells. Th2-cell-secreted IL-4 and Th2 cells costimulate naïve B cells for class-switch recombination and maturation of antigen-specific B cells [6]. Furthermore, we demonstrated that LF directly influenced DC-induced Th2 cell responses in OVA-induced asthma (Figure 6). Thus, we propose that the protective mechanism of LF in asthma may be through inhibition of the Th2 cell immune response in asthma (Figure 7).

In our previous studies, we demonstrated that LF reduced hyperoxia-induced lung injury and fibrosis in mice and attenuated lipopolysaccharide (LPS)-induced inflammation in human nasal epithelial cells [36,37,38]. Other studies also demonstrated that LF could ameliorate allergen- or pollutant-induced asthma [30,31,39]. However, administration of LF via intranasal instillation in mite-allergen-hypersensitive NC/Nga mice induced respiratory inflammation and asthmatic symptoms [40]. These studies suggest that the routes of LF administration might have different effects in asthma. In this study, the routes of OVA sensitization and LF treatment were intraperitoneal injection and oral administration, respectively.

## 4. Materials and Methods

### 4.1. Animals and Experimental Design

Eight-week-old male BALB/c mice (*n* = 20) and C57BL/6 mice (*n* = 5) were obtained from the National Laboratory Animal Center (Taipei, Taiwan), and OT-II TCR transgenic mice were purchased from Jackson Laboratory (Bar Harbor, ME, USA), and they were all housed under controlled temperature, humidity, and light in a specific pathogen-free central animal facility of National Chung Hsing University. All animals had free access to solid rodent chow food and water in facilities with a 12 h light and dark cycle and a temperature of 23 ± 2 °C. Mice were randomly divided into four groups according to treatment: normal control (NC), ovalbumin (OVA) (Thermo Fisher Scientific, Inc., Waltham, MA, USA)-sensitized, OVA-sensitized with a low dose of lactoferrin (100 mg/kg) (Sigma-Aldrich Co., St. Louis, MO, USA) treatment, and OVA-sensitized with a high dose of lactoferrin (300 mg/kg) treatment. Control group: mice received once-daily oral deionic water as a negative control. OVA-sensitized group: animals were immunized by intraperitoneal injection of 20 μg OVA with 2% alum in a total volume of 200 μL on the 1st, 7th, and 14th days; OVA/lactoferrin groups: animals were immunized and challenged following the sensitized OVA protocol and were also treated once daily by oral administration with 100 or 300 mg/kg lactoferrin. After lactoferrin treatment, the mice were challenged by intranasal instillation with 5% OVA for 30 min on the 21st to 27th day after the start of the sensitization period. The AHR of each mouse was measured as described below on the 32nd day, and the mice were sacrificed the next day using CO_2_ (air displacement rate, 30% of the chamber volume/min).

### 4.2. Measurement of AHR via Conscious Unrestrained Whole-Body Plethysmography

On the 32nd day, AHR was monitored by stimulation with aerosolized methacholine (Mch) (Sigma-Aldrich Co., Darmstadt, Germany) using a whole-body plethysmograph (Buxco; Harvard Bioscience, Inc., Holliston, MA, USA) [41]. Mice were first exposed to aerosolized PBS (0.8% NaCl, 0.02% KH_2_PO_4_, 0.115% Na_2_HPO_4_, 0.02% KCl; pH = 7.4) and then challenged with a series of aerosolized Mch doses from 3.125 to 50 mg/mL by an ultrasonic nebulizer and the mean outcome values for responses were collected over a 5 min period.

### 4.3. Bronchoalveolar Lavage Fluid (BALF) Analysis

The mice were sacrificed on the 33rd day and a needle was inserted into the trachea and used to slowly inject 1 mL of ice-cold PBS. The obtained BALF was centrifuged at 3420× *g* for 5 min to obtain the supernatant, which was then stored at −80 °C. Cytokine levels in BALF supernatants, including IL-4, IL-5, IL-13, and TNF-α, were assessed by ELISA kits (Thermo Fisher Scientific, Inc.). In addition, cells from the BALF were resuspended in 200 μL of PBS, and the total cell counts were calculated by mixing with 0.4% trypan blue (Sigma-Aldrich Co., St. Louis, MO, USA) using a hemocytometer [42]. Another part of BALF cells was used to prepare CytospinR slides (Thermo Shandon, Inc., Kalamazoo, MI, USA) with a Diff-Quik Staining reagent (Cat. 38721; Sysmex, Kobe, Japan). The number of monocytes, eosinophils, neutrophils, and lymphocytes in a total of 200 cells was calculated according to morphology.

### 4.4. Pulmonary Histology Scoring

The dissected lung was fixed for 15 min in 10% buffered formalin diluted in PBS at pH 7.2. The paraffin-embedded tissues were cut into 5-μm-thick sections. For evaluation of inflammatory cell infiltration and goblet cell hyperplasia, paraffin sections were stained with hematoxylin and eosin (H&E) reagents (Sigma-Aldrich Co., St. Louis, MO, USA) and analyzed by light microscopy (×200 magnification) for histopathological assessment [43,44]. For quantification of goblet cell hyperplasia, slides were examined in a double-blind setting according to a semiquantitative scoring system [45]. Briefly, the pathological changes were evaluated by 5-point scores (grade 0–4) according to the percentage of the goblet cells in the epithelium: 0 (no goblet cells), 1 (<25%), 2 (25–50%), 3 (51–75%), and 4 (>75%).

### 4.5. Serum OVA-Specific IgE and IgG1 Detection

A 96-well plate was precoated with 200 μg OVA in 0.1 M NaHCO_3_ at pH 9.6 of coating solution at 4 °C overnight. After PBS washes and blocking with 1% BSA (Sigma-Aldrich Co.), sera were loaded into wells and incubated at 4 °C overnight. After PBS washes, the wells were incubated with horseradish peroxidase (HRP)-conjugated rat anti-mouse IgE (GeneTex, Inc., Hsinchu, Taiwan) or goat anti-mouse IgG1 (Abcam, Cambridge, UK) antibodies at 4 °C overnight. After PBS washes, 3,3′,5,5′-tetramethylbenzidine (TMB) (Sigma) was added to the wells and incubated at room temperature for 15 min. The absorbance at 450 nm was measured using an ELISA reader (Tecan Trading AG, Männedorf, Switzerland).

### 4.6. OVA-Specific Splenocyte Responses

Spleens were harvested on the 33rd day and a single-cell suspension of splenocytes was generated by passing 50-mesh stainless mesh. For OVA-specific cell proliferation and cytokine expression, the cells (2 × 10^6^ cells/mL) were treated with OVA (100 μg/mL) in complete RPMI-1640 medium (Thermo Fisher Scientific, Inc.) and incubated for 72 h. The cells were collected and centrifuged at 1000× *g* for 15 min at 4 °C. The supernatant was collected for the detection of specific cytokine expression. The levels of IL-10, IL-4, and IFN-γ were evaluated by ELISA kits (Thermo Fisher Scientific, Inc.). For intracellular cytokine detection, splenocytes were incubated with OVA (50 μg/mL) for 72 h. Brefeldin A (BFA) (BD Biosciences, San Jose, CA, USA) was added for 4 h before harvesting, and then the cells were washed twice with PBS and stained with a PerCP-Cy5.5-conjugated anti-mouse CD4 antibody (BD Biosciences). Cells were fixed with fixation buffer (Biolegend, San Diego, CA, USA) and permeabilized with wash buffer (Biolegend) before staining with PE-conjugated specific antibodies against murine IFN-γ, IL-4, and Foxp3 (BioLegend). Subsequently, all samples were analyzed by a BD Accuri™ 5 flow cytometer (BD Biosciences). The mean fluorescence intensity (MFI) from each sample was calculated using C6 Accuri system software (version 1.0.264.15, BD Biosciences).

### 4.7. Flow Cytometric Analysis of DC Surface Markers

On the 33rd day, the harvested splenocytes were fixed in 100 μL of fixation buffer (BioLegend) for 30 min at 4 °C and washed twice with 1 mL of wash buffer (PBS containing 0.1% BSA). After centrifugation at 400× *g* for 5 min at 4 °C, the cells were stained with specific antibodies, including FITC-conjugated anti-mouse CD11c, PE-conjugated anti-mouse CD86, and anti-mouse CD80 antibodies (Biolegend), for 30 min at 4 °C. The PE rat IgG2a,κ isotype control antibody (Biolegend) was used as a negative control [21]. The samples were acquired with a forward side scatter threshold of 80,000 and a live gate on CD11c-positive events by a BD Accuri™ 5 flow cytometer (BD Biosciences). The MFI of cells was calculated using BD Accuri™ C6 system software (version 1.0.264.15).

### 4.8. Isolation of CD11c^+^ DCs

CD11c^+^ DCs were positively enriched from splenocytes using mouse CD11c MicroBeads UltraPure and LS separation columns (Miltenyi Biotec., San Diego, CA, USA) according to the manufacturer’s instructions. The purity of CD11c-positive DCs was above 80% after staining with FITC-conjugated mouse anti-CD11c antibody. The MFI of cells was calculated using BD Accuri™ C6 system software.

### 4.9. Quantitative Reverse Transcription-PCR (qRT-PCR)

Total RNA was extracted from purified CD11c^+^ DCs using TRIzol^®^ reagent (Invitrogen, San Diego, CA, USA). cDNAs were reverse transcribed with 2 μg of total RNA with an MMLV Reverse Transcription kit (Protech, Sparks, NV, USA). The mRNA levels of IL-4, IL-12, and hypoxanthine guanine phosphoribosyl transferase 1 (HPRT1) were evaluated using a real-time PCR detection system (Bio-Rad, Hercules, CA, USA) with SYBR Green real-time PCR master mix (Toyobo, Osaka, Japan). The primer sets used for the qRT-PCR assay were as follows: IL-4: forward, 5′-TTTGAACGAGGTCACAGGAGAAG-3′ and reverse, 5′-AGGACGTTT GGCACATCCA-3′; IL-12: forward, 5′-GCCAGTACACCTGCCACAAA-3′ and reverse, 5′-TGTGGAGCAGCAGATGTGAGT-3′; and HPRT1: forward, 5′-GTTGGATAAGGCCAGA CTTTGTTG-3′ and reverse, 5′-GATTCAACTTGCGCCATCTTAGGC-3′. The relative expression levels of IL-4 and IL-12 were calculated by the 2^−ΔΔCt^ method. The relative expression level of HPRT1 was used as an internal control for normalization [22].

### 4.10. In Vitro DC Functional Assay

Bone marrow-derived dendritic cells (BMDCs) isolated from C57BL/6 mice were stimulated with 5 μg/mL OVA_323–339_ (OVAP_2_) (Echo Chemical Co., Taipei, Taiwan) and then treated with DMSO or lactoferrin (150 or 300 μg/mL) for 1 h at 37 °C. After treatment, the cells were collected and suspended in PBS. For detection of DC function, OVAP_2_-specific CD4^+^ T cells isolated from the splenocytes of OT-II TCR transgenic mice (kindly provided by Dr. Ching-Liang Chu at National Taiwan University, Taipei, Taiwan) underwent MACS cell separation according to the manufacturer’s protocol (Miltenyi Biotec., Bergisch Gladbach, Germany). The isolated DCs and CD4^+^ T cells were cocultured with the ratio of DCs:T at 1:10 for 72 h, and the proliferation of the cells was determined based on [^3^H] thymidine uptake. Moreover, supernatants were obtained from cocultures of DCs and CD4^+^ T cells for 72 h to detect the levels of IL-4, IL-10, and IFN-γ.

### 4.11. Statistical Analysis

All data are presented as the mean ± SEM and were analyzed with GraphPad Prism software (version 5.0; GraphPad Software, Inc., La Jolla, CA, USA). *p* values < 0.05 were considered to be significant in all experiments. The statistical significance was determined by Kruskal–Wallis and Dunn’s post hoc test, or one- or two-way ANOVAs and Tukey’s post hoc test.

## 5. Conclusions

In this study, our data demonstrated that LF exhibited protective effects in ameliorating AHR, as well as lung inflammation and damage, in reducing the expression of Th2 cytokines and the secretion of allergen-specific antibodies, in influencing the functions of DCs, and in decreasing the level of Th2 immune responses in a BALB/c mouse model of OVA-induced allergic asthma. There are many studies about the benefits of LF such as anti-inflammation, for injury in human nasal epithelial cells, and for airway-hyperresponsiveness stimulated by allergens. In our finding, oral administration of LF in mice can ameliorate OVA-induced asthma. This finding indicates that LF could easily be ingested into the body for moderating asthma. Moreover, with the mature and low-cost manufacturing process of LF purification from milk protein, LF may be a satisfactory compound for airway care. These results suggest that LF may be an alternative therapeutic strategy for asthma therapy.

## Data Availability

All data generated or analyzed during this study are included in this published article.

## Supplementary Materials

The following supporting information can be downloaded at: https://www.mdpi.com/article/10.3390/ijms232214185/s1.
