# Supplementary material for: Lactoferrin Ameliorates Ovalbumin-Induced Asthma in Mice through Reducing Dendritic-Cell-Derived Th2 Cell Responses
Source: Int J Mol Sci. 2022 Nov 16;23(22):14185. doi: 10.3390/ijms232214185 (PMC9696322; doi:10.3390/ijms232214185)
Supplement: Supplementary file 1 [file ijms-23-14185-s001.zip › ijms-2001159-supplementary.pdf]

Supplementary Figure S1.

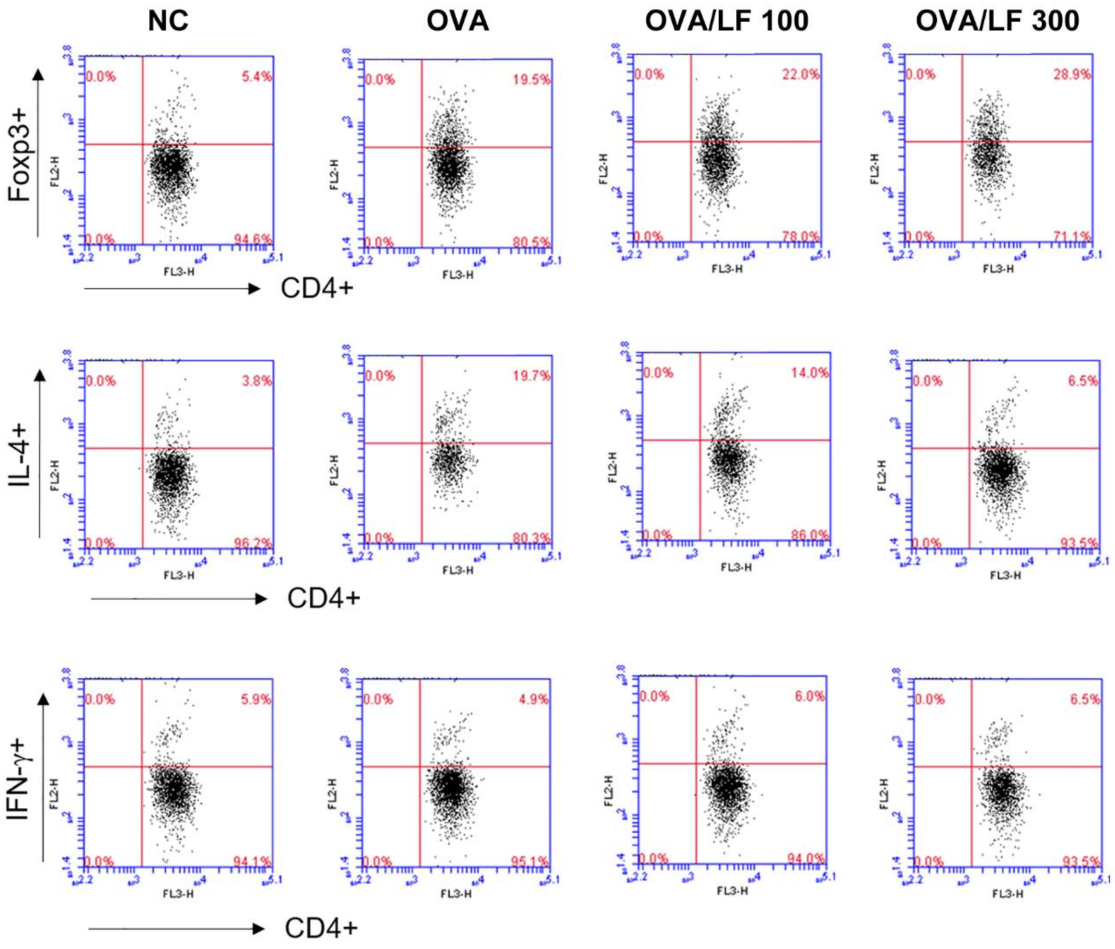

Supplementary Figure S1. The expression levels of Foxp3^{+}CD4^{+} Treg cells, IL-4^{+}CD4^{+} Th2 cells, and IFN-γ^{+}CD4^{+} Th1 cells in the splenocyte population. The levels of Foxp3, IL-4, IFN-γ, and CD4 were evaluated with specific antibodies by flow cytometry. NC: Normal control; OVA: ovalbumin; OVA/LF 100: OVA + lactoferrin 100 mg/kg; OVA/LF 300: OVA + lactoferrin 300 mg/kg.

Supplementary Figure S2.

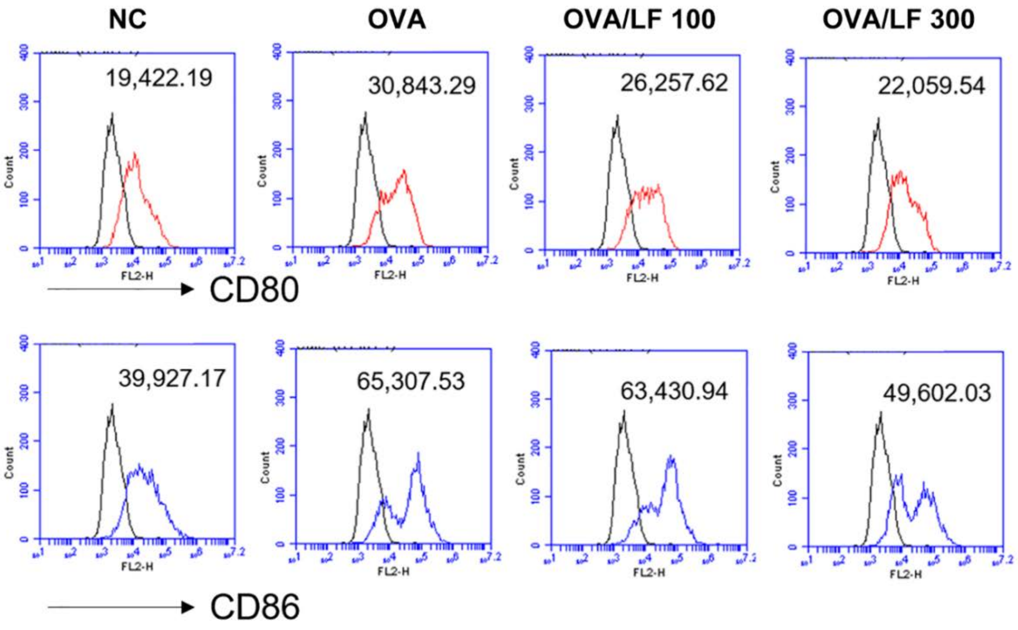

Supplementary Figure S2. The expression levels of CD80$^+$CD11c$^+$ and CD86$^+$CD11c$^+$ DCs in the splenocyte population. The levels of CD80 and CD86 were evaluated with specific antibodies by flow cytometry. NC: Normal control; OVA: ovalbumin; OVA/LF 100: OVA + lactoferrin 100 mg/kg; OVA/LF 300: OVA + lactoferrin 300 mg/kg.
